# Circulating tumor cells predict survival benefit from chemotherapy in patients with lung cancer

**DOI:** 10.18632/oncotarget.11707

**Published:** 2016-08-30

**Authors:** Zhuo-Xuan Wu, Zhen Liu, Han-Ling Jiang, Hong-Ming Pan, Wei-Dong Han

**Affiliations:** ^1^ Department of Medical Oncology, Sir Run Run Shaw Hospital, College of Medicine, Zhejiang University, Hangzhou, Zhejiang, China; ^2^ Department of Respiratory Medicine, Sir Run Run Shaw Hospital, College of Medicine, Zhejiang University, Hangzhou, Zhejiang, China

**Keywords:** circulating tumor cells, lung cancer, chemotherapy, tumor response, prognosis

## Abstract

**Background:**

This meta-analysis was to explore the clinical significance of circulating tumor cells (CTCs) in predicting the tumor response to chemotherapy and prognosis of patients with lung cancer.

**Methods:**

We searched PubMed, Embase, Cochrane Database, Web of Science and reference lists of relevant articles. Our meta-analysis was performed by Stata software, version 12.0, with a random effects model. Risk ratio (RR), hazard ratio (HR) and 95% confidence intervals (CI) were used as effect measures.

**Results:**

8 studies, including 453 patients, were eligible for analyses. We showed that the disease control rate (DCR) in CTCs-negative patients was significantly higher than CTCs-positive patients at baseline (RR = 2.56, 95%CI [1.36, 4.82], *p* < 0.05) and during chemotherapy (RR = 9.08, CI [3.44, 23.98], *p* < 0.001). Patients who converted form CTC-negative to positive or persistently positive during chemotherapy had a worse disease progression than those with CTC-positive to negative or persistently negative (RR = 8.52, CI [1.66, 43.83], *p* < 0.05). Detection of CTCs at baseline and during chemotherapy also indicated poor overall survival (OS) (baseline: HR = 3.43, CI [2.21, 5.33], p<0.001; during chemotherapy: HR = 3.16, CI [2.23, 4.48], *p* < 0.001) and progression-free survival (PFS) (baseline: HR = 3.16, 95%CI [2.23, 4.48], *p* < 0.001; during chemotherapy: HR = 3.78, CI [2.33, 6.13], *p* < 0.001).

**Conclusions:**

Detection of CTCs in peripheral blood indicates poor tumor response to chemotherapy and poor prognosis in patients with lung cancer.

## BACKGROUND

Lung cancer is the most commonly diagnosed cancer, as well as the leading cause of death in cancer patients worldwide [1]. Five-year survival rates of non-small cell lung cancer (NSCLC) and small cell lung cancer (SCLC) are less than 15% and 5%, respectively [2, 3]. Standard chemotherapy has greatly improved the cure rate. High-resolution imaging is the common approach to evaluating the treatment effect according to the Response Evaluation Criteria In Solid Tumors (RECIST) [4]. The sensitivity of RECIST is quite low when solid tumors change slightly. And we cannot detect the early tumor cell metastasis in this way, because these tumor cells are rare especially at the initial stage of localized lung cancer [5]. Therefore, a sensitive prognostic and predictive marker is urgently needed in lung clinical oncology.

Circulating tumor cells (CTCs) are cells migrating from solid tumors into the peripheral blood, which leads to the development of distant metastases [6–8]. The notion of CTCs in the peripheral blood was first raised by Ashworth in 1869 [9] and was demonstrated by Engell in 1955 [10]. In recent years, with the rapid development of the CTCs detection methods, such as immunocytochemistry (ICC) [11], reverse-transcription polymerase chain reaction (RT-PCR) [12] and CellSearch System [13], the clinical significance of CTCs is revealed gradually. At the initial stage of disease, CTCs can predict the risk of metastasis and evaluate prognosis [7]. During therapy, CTCs may be used as a biomarker to evaluate the response to treatment and guide the best therapy strategy.

Several studies aiming at breast cancer [14], colorectal cancer [15] and melanoma [16] have reported that CTCs status can be considered as a sensitive marker to predict prognosis. The study by Huang et al. [17] showed that CTCs could be used as a surrogate biomarker to assess the response to chemotherapy in colorectal cancer patients. They found that CTCs-positive during chemotherapy implied poor disease control rate (DCR), poor overall survival (OS) and progression-free survival (PFS). The prognosis role of CTCs for lung cancer has been explored by Ma et al. [18] that CTCs-positive meant poor OS and PFS. However, the actual significance of CTCs for predicting the response to chemotherapy in lung cancer patients is still controversial. Whether CTCs can be considered as a sensitive maker for the tumor response to chemotherapy needs to be verified.

Thus, this comprehensive meta-analysis was conducted to explore the therapeutic evaluation value of CTCs status for patients with lung cancer. Specifically, we assessed the relationship between CTCs status (positive *vs*. negative) and tumor response to chemotherapy according to RECIST. Furthermore, we analyzed the relationship between the conversion status of CTCs and tumor response to chemotherapy. In addition, the OS and PFS were assessed.

## RESULTS

### Baseline study characteristics

**Figure 1:**
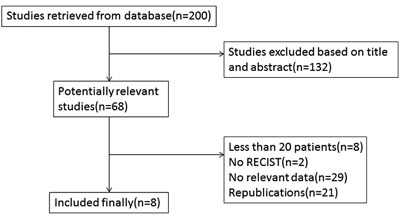
Flow chart of selecting eligible studies

Two hundred studies were initially identified in the systematic literature search. Only 8 studies were finally eligible for analysis (Figure 1). We excluded 192 studies following selection criteria. One hundred and thirty-two studies were excluded after screening titles and abstracts. Based on titles and abstracts, we excluded 5 reviews, 5 cases, 8 studies about circulating endothelial cells (CECs) instead of CTCs, 3 studies without chemotherapy, 21 studies exploring prognosis only, and others just making CTCs as a simple data without any relationship to tumor response during chemotherapy. During the 68 potential relevant studies, eight studies were excluded because they had less than 20 patients. Two studies that assessed tumor response to chemotherapy not according to the RECIST were excluded. Twenty-nine studies lacked the outcomes of interest. Twenty-one studies were replications.

All 8 studies included 453 eligible lung cancer patients. The sample size ranged from 30 to 101 patients (median sample size: 53; mean: 57). The studies were conducted in 4 countries (the United Kingdom, China, Japan and Netherlands) and published between 2009 and 2014. In the matter of detection methods, three studies detected CTCs by the means of CellSearch [19–21]. Two studies applied RTQ-PCR [22, 23]. Other two studies used CD45-FISH [24, 25]. The last study detected CTCs by the means of OBP-401 assay [26]. In the matter of detection time, all studies detected the CTCs both at baseline and during chemotherapy [19–26]. Two studies showed the relationship between tumor response to chemotherapy and the conversion of CTCs during chemotherapy [24, 25]. The main baseline characteristics were summarized in Table 1. The quality of eligible studies was summarized in Table 2.

**Table 1:** Baseline characteristics of the eligible studies in our meta-analysis

Reference	Number (M/F)	Rate(+)	S/NS	Sample time	CHT before and after ST	Age mean±SD/median (range)	Detection method	Follow-up mean±SD/median (range)	Tumor stage	Outcomes	Country	Surgery
Igawa 2014	30(28/2)	30%	30/0	Baseline	NO/YES	69(51-85)	OBP-401 assay	12	LD, ED	RECIST\OS\PFS	Japan	NR
	29	NR	29/0	Cycle 2	YES/YES	NR	OBP-401 assay	NR	LD, ED	RECIST\OS\PFS	Japan	NR
	NR	NR	NR	Cycle 3, 4, Progressive disease point	YES/YES	NR	OBP-401 assay	NR	LD, ED	RECIST\OS\PFS	Japan	NR
Du 2014	78(49/29)	69.2%	0/78	Baseline	NO/YES	62(43-74)	RTQ-PCR	11.8±5.2	IIIB, IV	RECIST\OS\PFS	China	NR
	78(49/29)	52.6%	0/78	Cycle 1	YES/YES	NR	RTQ-PCR	NR	IIIB, IV	RECIST\OS\PFS	China	NR
	75(47/28)	50.7%	0/75	Cycle 3	YES/YES	NR	RTQ-PCR	NR	IIIB, IV	RECIST\OS\PFS	China	NR
Chen 2014	50(35/15)	84%	11/39	Baseline	NO/YES	59(30-81)	CD45-FISH	6	7 I/II, 18III, 25IV	RECIST\PFS	China	NR
	25	NR	NR	Cycle 1	YES/YES	NR	CD45-FISH	NR	NR	RECIST\PFS	China	NR
	25	NR	NR	Cycle 2	YES/YES	NR	CD45-FISH	NR	NR	RECIST\PFS	China	NR
Shi 2013	55(36/19)	78.2%	55/0	Baseline	NO/YES	59(41-75)	RTQ-PCR	26	LD, ED	RECIST\OS\PFS	China	NO
	55(36/19)	32.7%	55/0	Cycle 1	YES/YES	NR	RTQ-PCR	NR	LD, ED	RECIST\OS\PFS	China	NO
	52	28.8%	52/0	Cycle 3	YES/YES	NR	RTQ-PCR	NR	LD, ED	RECIST\OS\PFS	China	NO
Hirose 2012	33(23/20)	36.4%	0/33	Baseline	NO/YES	64(46-74)	CellSearch	12	IV	RECIST\MST\PFS	Japan	NO
	27	NR	0/27	Cycle 2	YES/YES	NR	CellSearch	NR	IV	RECIST\MST\PFS	Japan	NO
Hiltermann 2012	59(35/24)	73%	59/0	Baseline	NO/YES	64(47-84)	CellSearch	9.3(0.2-47.5)	LD, ED	RECIST\OS\PFS	Netherlands	NR
	37	NR	37/0	Cycle 1	YES/YES	NR	CellSearch	NR	LD, ED	RECIST\OS\PFS	Netherlands	NR
	34	NR	34/0	Cycle 4	YES/NO	NR	CellSearch	NR	LD, ED	RECIST\OS\PFS	Netherlands	NR
Krebs 2011	101(54/47)	21%	0/101	Baseline	NO/YES	67(43-84)	CellSearch	5.4±4.1	IIIA,IIIB,IV	RECIST\OS\PFS	UK	NR
	70	7.1%	0/70	Cycle 1	YES/YES	NR	CellSearch	NR	IIIA, IIIB, IV	RECIST\OS\PFS	UK	NR
Wu 2009	47	63.8%	13/34	Baseline	NO/YES	NR	Cytelligen	NR	3 I/II, 22III, 22IV	RECIST	China	NO
	12	50%	3/9	Cycles 2	YES/YES	NR	Cytelligen	NR	NR	RECIST	China	NO

NOTE, M/F: Male/Female; S/NS: Small cell lung cancer/Non-small cell lung cancer; CHT: Chemotherapy before and after sampling time; SD: Standard deviation; Rate (+): Rate of CTCs-positive patients, n/N (%); LD: Limited disease stage; ED: Extensive disease stage; OS: Overall survival; PFS: Progression-free survival; MST: Median survival time; RECIST: Response evaluation criteria in solid tumors; RTQ-PCR: Real time quantity polymerase chain reaction; NR: Not reported.

**Table 2:** The assessment of the risk of bias by the Newcastle-Ottawa scale

Study	Selection(0-4)				Comparability(0-2)		Outcome(0-3)			Total
	REC	SNEC	AE	DO	SC	AF	AO	FU	AFU	
Igawa 2014	1	0	1	1	0	1	1	1	1	7
Du 2014	1	1	1	1	0	1	1	1	1	8
Chen 2014	1	1	1	1	0	1	1	1	0	7
Shi 2013	1	1	1	1	0	1	1	1	1	8
Hirose 2012	1	0	1	1	0	1	1	1	0	6
Hilterman 2012	1	0	1	1	0	1	1	1	0	6
Krebs 2011	1	0	1	1	0	1	1	0	0	5
Wu 2009	1	1	1	1	0	1	1	0	0	6

NOTE. REC: representativeness of the exposed cohort; SNEC: selection of the non-exposed cohort; AE: ascertainment of exposure; DO: demonstration that outcome of interest was not present at start of study; SC: study controls for age, sex; AF: study controls for any additional factors (chemoradiotherapy, curative resection); AO: assessment of outcome; FU: follow-up long enough(6 months) for outcomes to occur; AFU: adequacy of follow-up of cohorts (≥90%). ‘1′ means that the study is satisfied the item, and ‘0′ means the opposite situation.

### Relationship between CTCs and tumor response to chemotherapy

**Figure 2:**
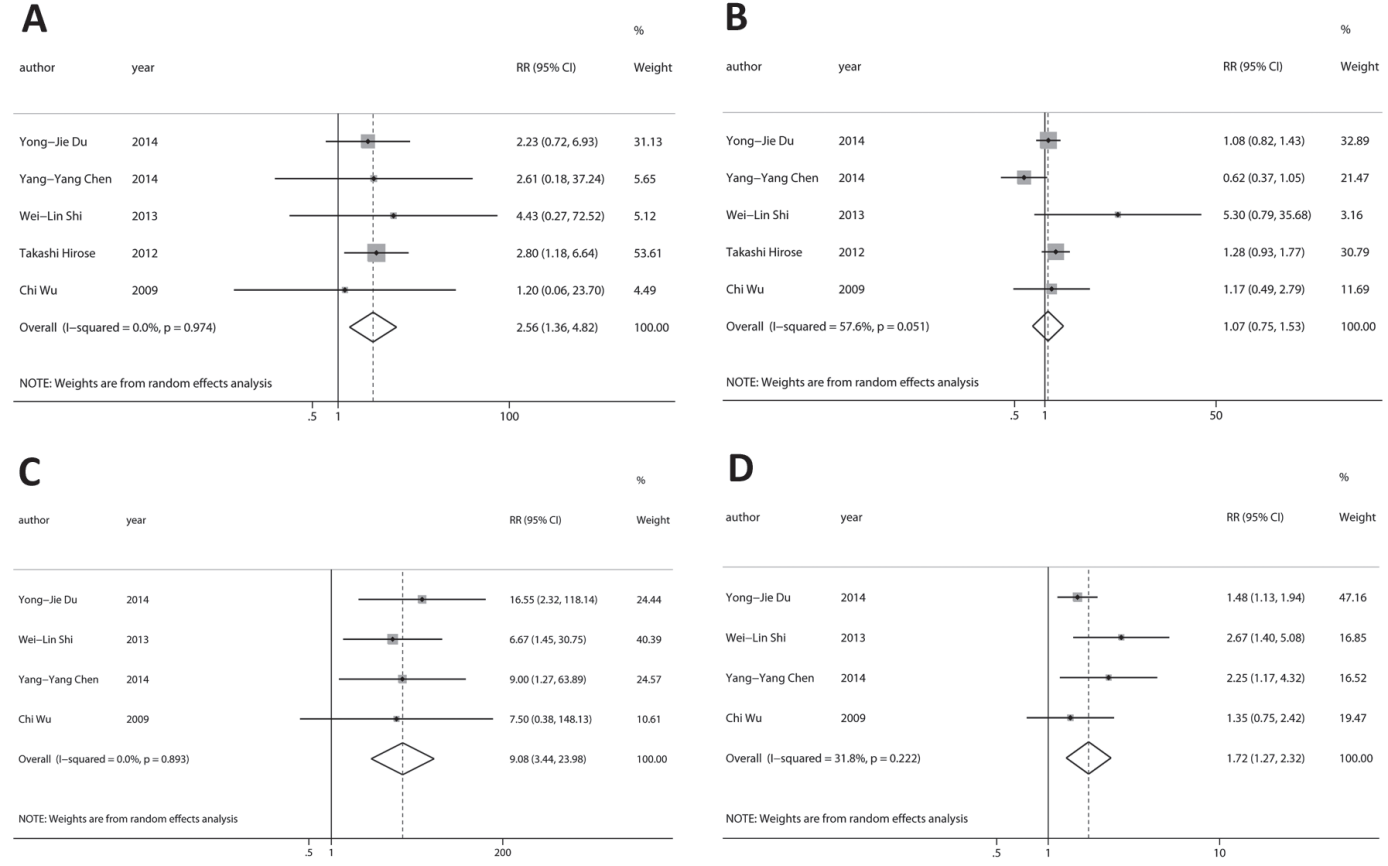
Relationship between CTCs and tumor response to chemotherapy At baseline, **A.,** the DCR of CTCs-negative was significantly higher than CTCs-positive, while **B.,** there was not statistical difference about the ORR between CTCs-negative and CTCs-positive. During chemotherapy, **C.,** the DCR and **D.,** the ORR of CTCs-negative was significantly higher than CTCs-positive.

At baseline, the DCR of CTCs-negative was significantly higher than CTCs-positive (Figure 2A; RR = 2.56, 95%CI [1.36, 4.82], *p* < 0.01, I^2^ = 0.0%), while there was no statistical difference about the objective response rate (ORR) between CTCs-negative and CTCs-positive (Figure 2B; RR = 1.07, 95%CI [0.75, 1.53], *p* > 0.05, I^2^ = 57.6%). Sensitivity and specificity of CTCs-positive for RECIST-based disease progression (PD) were 90% (95%CI [67%, 97%]) and 35% (95%CI [18%, 57%]) respectively. Positive and negative likelihood ratios were 1.4 (95%CI [1.1, 1.8]) and 0.3 (95%CI [0.11, 0.88]) respectively. The AUROC was 0.72 (95%CI [0.68, 0.76]). Diagnostic odds ratio was 4.5 (95%CI [1.4, 14.6]).

During chemotherapy, the DCR of CTCs-negative was significantly higher than CTCs-positive (Figure 2C; RR = 9.08, 95%CI [3.44, 23.98], *p* < 0.001, I^2^ = 0.0%), and the ORR of CTCs-negative was also significantly higher than CTCs-positive (Figure 2D; RR = 1.72, 95%CI [1.27, 2.32], *p* < 0.001, I^2^ = 31.8%). Sensitivity and specificity of CTCs-positive for RECIST-based disease nonresponse (SD +PD) were 56% (95%CI [46%, 65%]) and 86% (95%CI [75%, 93%]) respectively. Positive and negative likelihood ratios were 4.0 (95%CI [2.1, 7.6]) and 0.51 (95%CI [0.40, 0.65]) respectively. The AUROC was 0.86 (95%CI [0.82, 0.89]). Diagnostic odds ratio was 8 (95%CI [4, 18]).

The level of CTCs in peripheral blood is dynamic. To get more details, the chemotherapy cycle (cycle 1, cycle 2, cycle 3) was also analyzed individually (Table 3 and Figure S3). We found that CTCs detected after cycle 1 had no relationship with tumor response to chemotherapy in both DCR (Table 3; RR = 3.16, 95%CI [0.29, 34.14], *p* > 0.05, I^2^ = 86.8%) and ORR (Table 3; RR = 1.44, 95%CI [0.89, 2.31], *p* > 0.05, I^2^ = 59.3%). However, CTCs detected after cycle 2 and 3 showed the significant relationship with tumor response to chemotherapy in both DCR (Table 3; cycle 2: RR = 8.52, 95%CI [1.66, 43.83], *p* < 0.05, I^2^ = 0.0%; cycle 3: RR = 9.39, 95%CI [2.81, 31.39], *p* < 0.001, I^2^ = 0.0%) and ORR (Table 3; cycle 2: RR = 1.71, 95%CI [1.00, 2.91], *p* < 0.05, I^2^ = 32.6%; cycle 3: RR = 1.85, 95%CI [1.04, 3.30], *p* < 0.05, I^2^ = 65.6%). Especially, CTCs detected after cycle 3 had a smaller *p* value compared with cycle 2. It is likely that curative effect tends to be stable with treatments keeping on.

**Table 3:** Relationship between CTCs in different chemotherapy cycle and tumor response to chemotherapy

		Baseline and during chemotherapy				
				During chemotherapy		
	Any	Baseline	Any	Cycle 1	Cycle 2	Cycle 3
Disease control rate	3.72(1.86, 7.43) I^2^ = 49.9% *P* < 0.001	2.56(1.36, 4.82) I^2^ = 0.0% *P* < 0.01	5.43(1.60, 18.49) I^2^ = 71.1% *P* < 0.01	3.16(0.29, 34.14) I^2^ = 86.8% *P* > 0.05	8.52(1.66, 43.83) I^2^ = 0.0% *P* < 0.05	9.39(2.81,31.39) I^2^ = 0.0% *P* < 0.001
Objective response rate	1.37(1.11, 1.71) I^2^ = 59.7% *P* < 0.01	1.07(0.75, 1.53) I^2^ = 57.6% *p* > 0.05	1.59(1.26, 2.00) I^2^ = 40.8% *P* < 0.001	1.44(0.89, 2.31) I^2^ = 59.3% *p* > 0.05	1.71(1.00, 2.91) I^2^ = 32.6% *P* < 0.05	1.85(1.04, 3.30) I^2^ = 65.6% *P* < 0.05
HR for OS	3.83(2.49, 5.88) I^2^ = 2.2% *P* < 0.001	3.43(2.21, 5.33) I^2^ = 19.8% *P* < 0.001	/	3.83(2.49, 5.88) I^2^ = 7.9% *P* < 0.001	/	/
HR for FPS	3.34(2.58, 4.32) I^2^ = 0.0% *P* < 0.001	3.16(2.23, 4.48) I^2^ = 0.0% *P* < 0.001	/	3.78(2.33, 6.13) I^2^ = 11.5% *P* < 0.001	/	/

### Relationship between conversion status of CTCs and tumor response to chemotherapy

CTCs level of a patient detected at baseline and during chemotherapy was ordinarily different. All patients were divided into four groups according to the transformation of CTCs status. Group 1 consisted of patients who converted from CTCs-negative to CTCs-positive. Group 2 consisted of patients who were persistently CTCs-positive. Group 3 consisted of patients who converted from CTCs-positive to CTCs-negative. Group 4 consisted of patients who were persistently CTCs-negative.

**Figure 3:**
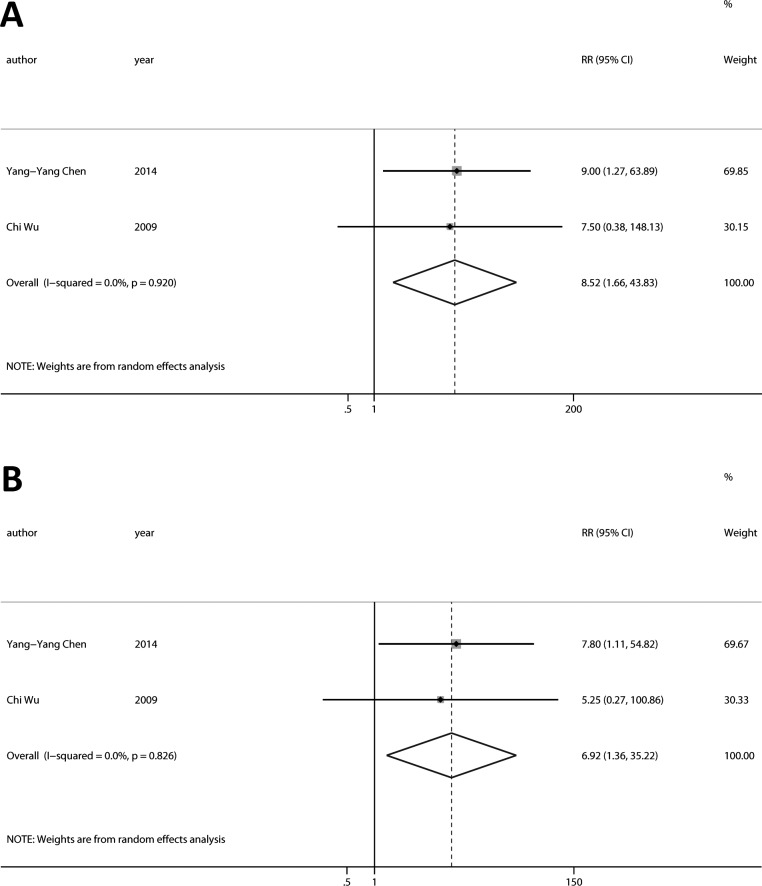
Relationship between conversion status of CTCs and tumor response to chemotherapy **A.** Group 1 and 2 had a significant disease progression compared with Group 3 and 4. **B.** Group 1 and 2 had a significant disease progression compared with Group 3. (Group 1: CTCs- to CTCs+; Group 2: persistently CTCs+; Group 3: CTCs+ to CTCs-; Group 4: persistently CTCs-).

Our results suggested that group 1 and 2 *vs*. Group 3 and 4 as well as *vs*. Group 3 had significant disease progression (Figure 3A; RR = 8.52, 95%CI [1.66, 43.83], *p* < 0.05, I^2^ = 0.0%; Figure 3B; RR = 6.92, 95%CI [1.36, 35.22], *p* < 0.05, I^2^ = 0.0%).

### Relationship between CTCs and survival outcomes (PFS and OS)

**Figure 4:**
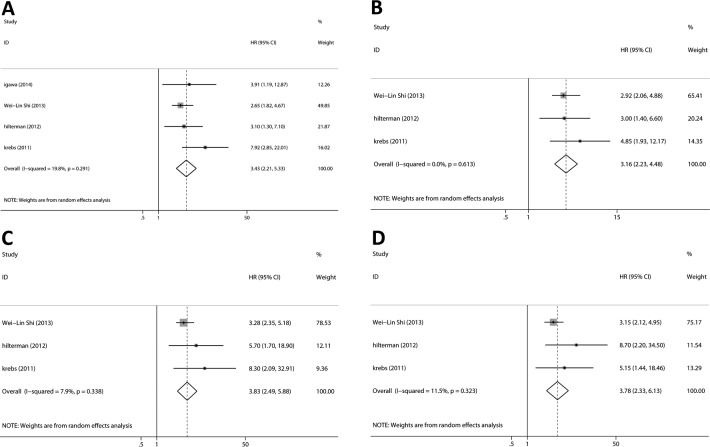
Relationship between CTCs and survival outcomes (PFS and OS) The pooled HRs suggested that CTCs-positive had significant **A.** poor OS at baseline, **B.** poor PFS at baseline, **C.** poor OS during chemotherapy and **D.** poor PFS during chemotherapy.

At baseline, HRs for OS were available in four studies. The pooled HRs suggested that CTCs-positive had a significant poor OS (Figure 4A; HR = 3.43, 95%CI [2.21, 5.33], *p* < 0.001, I^2^ = 19.8%). Three studies provided HRs for PFS, and the estimated pooled HRs indicated that CTCs-positive had a significant poor PFS (Figure 4B; HR = 3.16, 95%CI [2.23,4.48], *p* < 0.001, I^2^ = 0.0%).

During chemotherapy, HRs for OS were available in three studies. The pooled HRs suggested that CTCs-positive had a significant poor OS (Figure 4C; HR = 3.83, 95%CI [2.49, 5.88], *p* < 0.001, I^2^ = 7.9%). Three studies provided HRs for PFS, and the estimated pooled HRs indicated that CTC-positive had a significant poor PFS (Figure 4D; HR = 3.78, 95%CI [2.33, 6.13], *p* < 0.001, I^2^ = 11.5%).

### Evaluation of heterogeneity and publication bias

The heterogeneity of statistically significant results was less than 40%, which was acceptable according to Cochrane Handbook [27]. Publication bias was assessed with the funnel plot analyses, Egger's and Begg's tests (see Appendix 2 and Supplementary Figure S1.). Except the HRs for OS during chemotherapy, there was no significant publication bias. We used trim-and-fill analysis which indicated that there might be two missing or unpublished studies for OS during chemotherapy. The sensitivity analysis indicated that our results were not dominated by single study (see Supplementary Figure S2).

## DISCUSSION

The CTCs status was related to prognosis and response to chemotherapy in many tumors. Rahbari et al. [15] showed that detection of circulating tumor cells indicated poor prognosis in patients with colorectal cancer. Huang et al. [17] indicated that CTCs could be useful as a surrogate marker for the response to chemotherapy providing additional prognostic information to tumor radiographic imaging. Similar outcomes were also found in breast cancer [28, 29], melanoma [16] and prostate cancer [30]. In clinical researches, Li et al. [31] showed pERK/pAkt phenotyping in circulating tumor cells as a biomarker for sorafenib efficacy in patients with advanced hepatocellular carcinoma. Marin-Aguilera et al. [32] showed that the enumeration of circulating tumor cells in peripheral blood correlated with clinical outcome in castration-resistant prostate cancer. The single prognostic role of CTCs for lung cancer was also revealed [18, 33]. However, the role of CTCs in predicting the tumor response to chemotherapy in lung cancer patients remains unclear. Our findings indicate that detection of CTCs in peripheral blood predicts poor tumor response to chemotherapy and poor prognosis in patients with lung cancer. To our best knowledge, this is the first meta-analysis assessing the predictive and prognostic significance of CTCs in lung cancer patients treated with chemotherapy.

The tumor response to chemotherapy was assessed according to the RECIST guidelines, as radiographic image was the gold standard. In this study, we found that baseline CTCs status could predict DCR for the tumor response to chemotherapy (*p* < 0.01). And CTCs detected during chemotherapy were also significantly associated with both ORR and DCR (*p* < 0.001) for tumor response to chemotherapy. CTCs are cells migrating from solid tumors into the peripheral blood, which leads to the development of distant metastases [6–8, 34]. Thus, it is reasonable that CTCs-positive means the poor response.

The chemotherapy cycle (cycle 1, cycle 2, cycle 3) was analyzed individually. We found that CTCs in different cycles showed different relationship with tumor response to chemotherapy. Especially, CTCs in cycle 3 had a smaller p value compared with cycle 2. It is likely that curative effect tends to be stable with treatment keeping on. Given this, it is more reasonable that CTCs detected in the latter chemotherapy cycle, instead of the whole cycles, represented CTCs status during chemotherapy for the further exploration.

The level of CTCs in peripheral blood is dynamic. Matthew G. Krebs et.al [21] reported that NSCLC patients with reduction in CTCs number after one cycle of chemotherapy had a significantly better response than those with increased or unchanged CTCs number, indicating a potential use of CTCs as a surrogate end point to predict the efficacy of chemotherapy. Here, we found that the conversion of CTCs status was significantly associated with the tumor response to chemotherapy. Patients who converted from CTCs-negative to CTCs-positive or who were persistently CTCs-positive had a poorer DCR compared with patients who converted from CTCs-positive to CTCs-negative or who were persistently negative. This might be because that changes in CTCs level implied the change of tumor proliferative ability and chemotherapeutic sensitivity [35]. Also, the larger tumor would release more circulating tumor cells into peripheral blood, and effective chemotherapy restricted the CTCs. Thus, our analysis indicated that the “real-time” level of CTCs combined with radiographic imaging should be more sensitive for guiding individual chemotherapy. However, it is unreachable to make these factors for further explorations on primary tumor size and tumor proliferation now. We cannot get enough eligible studies, so that some subgroups had only one or none study, which was the biggest obstruction for us to get more conclusions.

In this meta-analysis, we also demonstrated that the level of CTCs detected at baseline and during chemotherapy were correlated with OS and PFS, which was consisted with previously published meta-analysis [18, 33]. However, many studies focusing on the prognostic role of CTCs for lung cancer were excluded from our analysis. That was because the key point of our meta-analysis was to explore the clinical significance of CTCs in predicting the tumor response to chemotherapy, thus the eligible studies must meet the criteria that the study had the data of the level of CTCs and patients' response to chemotherapy. Studies only exploring the prognosis were excluded. In this way, our analysis about the prognosis of CTCs in lung cancer was incomprehensive. It was understandable that the OS during chemotherapy had publication bias calculated by Egger's Test.

The heterogeneity of mainly statistically significant results was less than 40%, which was acceptable according to Cochrane Handbook [27]. Of course, there are also several heterogeneity of statistically significant results more than 40% (Table 3). As previously mentioned, it is necessary for us to know that CTCs in different cycles showed different relationship with tumor response to chemotherapy due to its dynamic property. However, it is more reasonable that CTCs detected in the last chemotherapy cycle instead of the whole cycles represented CTCs status during chemotherapy. No significant publication bias was found in the results except the OS during chemotherapy. The sensitivity analysis indicated that our results were not dominated by single study, confirming the stability of our results.

Due to the limited studies and patients included, there are several limitations of this meta-analysis. Firstly, studies in this field were relatively few and some subgroups had only one or none study. We could not perform deep subgroup analysis, such as response assessment time, detection methods, regions, primary tumor size, tumor proliferation and so on. We made a mixture of studies evaluating NSCLC and SCLC. Two studies rolling SCLC and NSCLC up into one were included. We cannot extract the SCLC and NSCLC data from these two studies, which was an obstruction for us to compare CTCs in SCLC to NSCLC, respectively. Secondly, although no significant publication bias was found, the limited number of studies would affect the statistical power, as well as the sensitivity. Large-scale, random controlled multicenter studies should be conducted to confirm these results. Thirdly, the definition of CTCs cut-off point was different between studies, which would affect the pooled statistical results. Despite these limitations, this is the first meta-analysis to assess the therapeutic prediction value of CTCs status for lung cancer patients treated with chemotherapy.

## CONCLUSIONS

In summary, detection of CTCs in peripheral blood indicates poor tumor response to chemotherapy and poor prognosis in patients with lung cancer.

## MATERIALS AND METHODS

### Search strategy

Several main databases were systematically searched without language, place and time restrictions (up to December, 2015): PubMed, Embase, Cochrane Database and Web of Science. The reference lists of 5 identified review articles were also checked manually for potentially relevant studies. The main terms we used in search were “Lung Neoplasms”, “Drug Chemotherapy” and “Neoplastic Cells, Circulating”. (See Appendix 1 for more search strategies details).

### Selection criteria

To be eligible, studies had to meet all of the following criteria: (1) analyzed patients number was not less than 20; (2) all patients enrolled were diagnosed with lung cancer; (3) chemotherapy as the only treatment; (4) CTCs was one of the evaluation indexes for the efficacy of chemotherapy; (5) CTCs were detected at baseline and during chemotherapy (before the chemotherapy as baseline, after chemotherapy as during chemotherapy); (6) the specific circulating tumor cell or its DNA was detected on behalf of the CTCs; (7) tumor response to chemotherapy was assessed according to the RECIST (Response Evaluation Criteria In Solid Tumors) 1.1 guidelines (complete response as CR, partial response as PR, stable disease as SD, progressive disease as PD) [36]; (8) the study had to explore the association between the level of CTCs and patients' response to chemotherapy (CR, PR, SD and PD).

### Data extraction

Two reviewers (Z. X. Wu and Z. Liu) independently screened each study lists and extracted the following data from eligible studies: first author, publication time, population characteristics (the number of analyzed patients, age, tumor stage, the number of patients with SCLC or NSCLC), rate of CTCs-positive patients, sampling time (baseline and during chemotherapy), detection method, duration of follow-up, response to chemotherapy (CR, PR, SD and PD) and prognostic indexes (OS and FPS). All disagreements were resolved by discussions.

### Quality assessment

The quality of the selected studies was evaluated by the Newcastle-Ottawa Scale (NOS) criteria [37]. Publication bias was assessed with the funnel plot analyses, Egger's and Begg's tests [38, 39]. All disagreements were resolved by discussions.

### Statistical analysis

The hazard ratios (HRs), odds ratios (ORs) and risk ratios (RRs) were extracted as evaluation indexes for response to chemotherapy and prognosis. If the HRs and their related 95% confidence intervals (95% CIs) or P values were not provided in the article directly, we calculated HRs by the method reported by Jayne F. Tierney [40] using data extracted from original articles. We took the CTCs level of last cycle as “during chemotherapy” if patients experienced more than one cycle of chemotherapy.

HR >1 means a worse outcome in the CTCs-positive group compared with the CTCs-negative group. All HRs were pooled together by Stata software (version 12.0) (Stata Corp, College Station, TX, USA). Given heterogeneity, we used a random effect analysis model, which provided more conservative estimates than the fixed effect analysis model [41]. Statistical heterogeneity among studies was assessed with the Cochran Q test and I^2^ statistics [42]. Sensitivity and specificity were calculated using a bivariate mixed-effects regression model [43, 44]. A sensitivity analysis was performed to assess the consistency of the results.

We evaluated the relationship between the CTCs level and the response to chemotherapy, while radiographic imaging was the gold standard. On the other hand, we explored the possibility of CTCs level as a biomarker in predicting the response (CR+PR) or the disease control rate (CR+PR+SD). A P value was set at 0.001, 0.01 and 0.05. CI was set at 95%.

## SUPPLEMENTARY MATERIAL FIGURES AND TABLES


