# Supplementary material for: Circulating tumor cells predict survival benefit from chemotherapy in patients with lung cancer
Source: Oncotarget. 2016 Aug 30;7(41):67586–96. doi: 10.18632/oncotarget.11707 (PMC5341898; doi:10.18632/oncotarget.11707)
Supplement: Supplementary file 1 [file oncotarget-07-67586-s001.pdf]

Circulating tumor cells predict survival benefit from chemotherapy in patients with lung cancer

Supplementary Materials

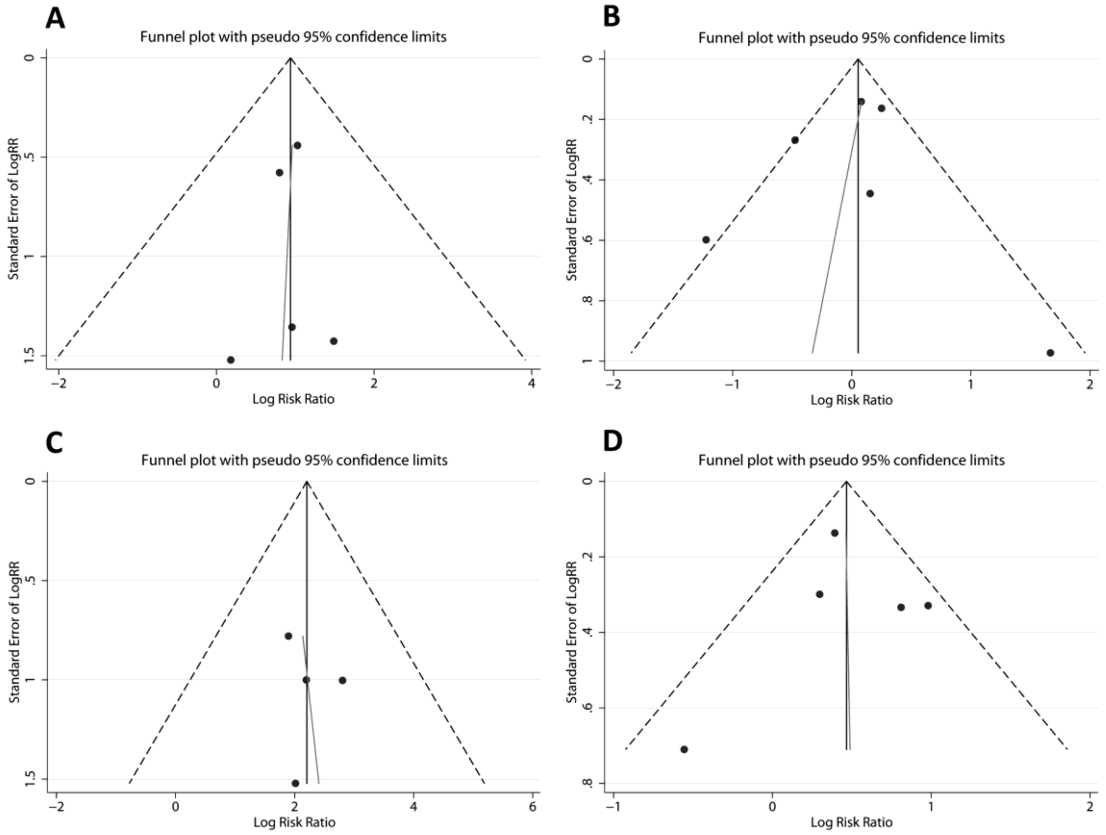

Supplementary Figure S1. Qualitative analyses of publication bias by Funnel plot. The publication bias of **A.** DCR at baseline, **B.** ORR at baseline, **C.** DCR during chemotherapy and **D.** ORR during chemotherapy.

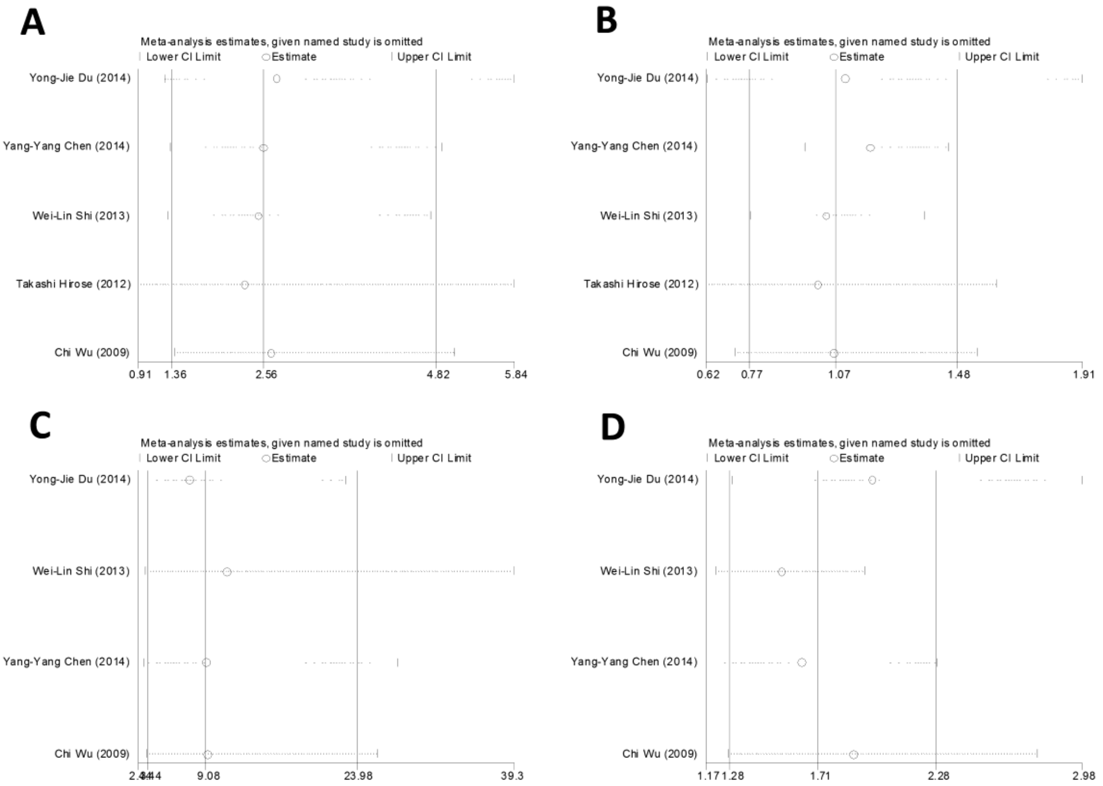

Supplementary Figure S2. Qualitative analyses of sensitivity analysis based on leave-one-out approach. The sensitivity analysis of **A.** DCR at baseline, **B.** ORR at baseline, **C.** DCR during chemotherapy and **D.** ORR during chemotherapy.

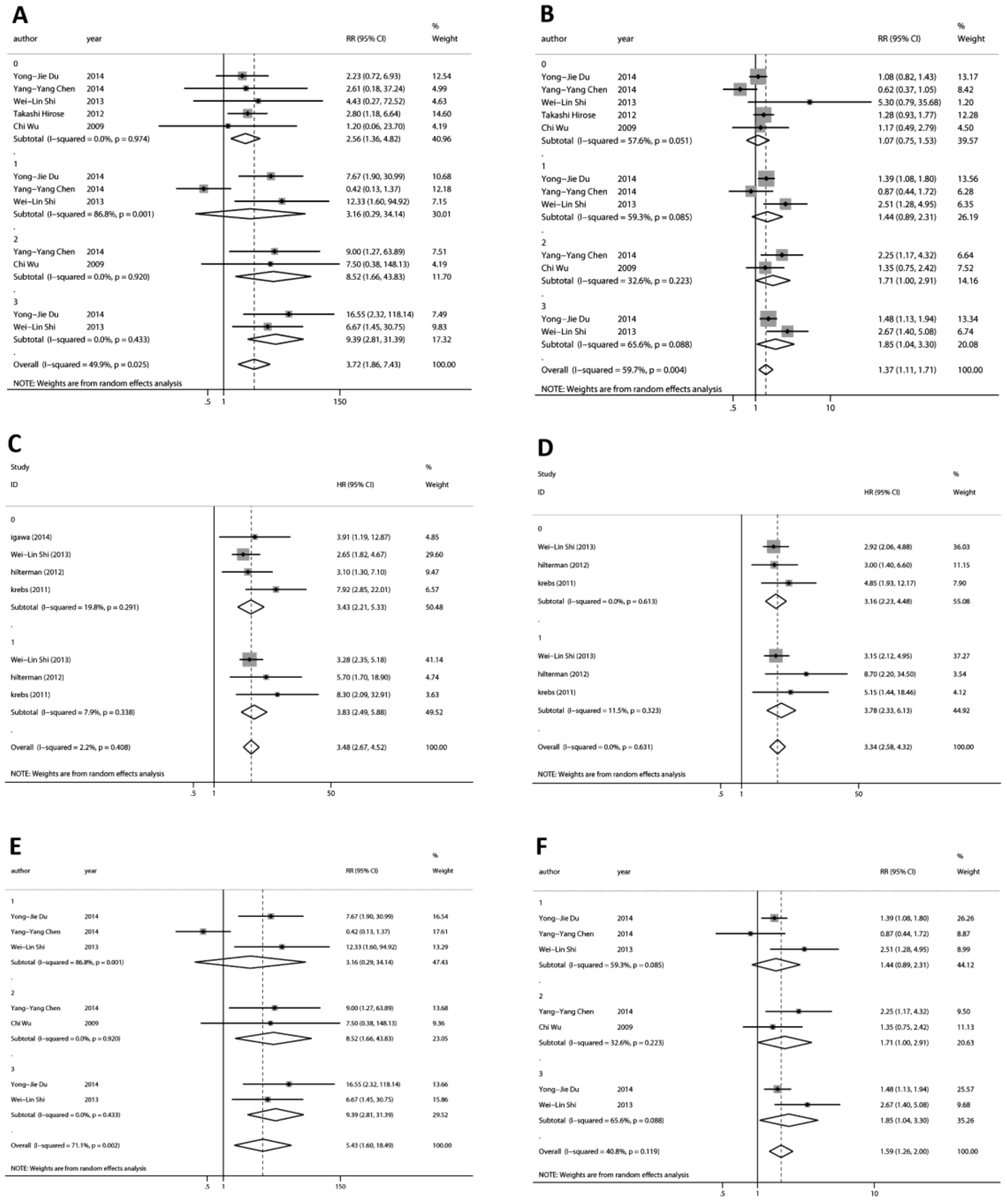

Supplementary Figure S3. Relationship between CTCs in different chemotherapy cycle and tumor response to chemotherapy. At baseline (0), chemotherapy cycle 1, 2, and 3, **A.** the DCR, **B.** the ORR, **C.** the OS and **D.** the PFS of CTCs-negative compared with CTCs-positive. In the chemotherapy cycle 1, 2, and 3, **E.** the DCR and **F.** the ORR of CTCs-negative compared with CTCs-positive.

Appendix 1. Search Strategies

PubMed

Searched up to December, 2015

No.	Searches	Results
1	"Lung Neoplasms"[Mesh]	188044
2	((((((((((((((((Pulmonary Neoplasms[Title/Abstract]) OR Neoplasms, Lung[Title/Abstract]) OR Lung Neoplasm[Title/Abstract]) OR Neoplasm, Lung[Title/Abstract]) OR Neoplasms, Pulmonary[Title/Abstract]) OR Neoplasm, Pulmonary[Title/Abstract]) OR Pulmonary Neoplasm[Title/Abstract]) OR Lung Cancer[Title/Abstract]) OR Cancer, Lung[Title/Abstract]) OR Cancers, Lung[Title/Abstract]) OR Lung Cancers[Title/Abstract]) OR Pulmonary Cancer[Title/Abstract]) OR Cancer, Pulmonary[Title/Abstract]) OR Cancers, Pulmonary[Title/Abstract]) OR Pulmonary Cancers[Title/Abstract]) OR Cancer of the Lung[Title/Abstract]) OR Cancer of Lung[Title/Abstract]	224585
3	1 OR 2	272754
4	"Drug Therapy"[Mesh]	1127889
5	((((((Therapy, Drug[Title/Abstract]) OR Drug Therapies[Title/Abstract]) OR Therapies, Drug[Title/Abstract]) OR Chemotherapy[Title/Abstract]) OR Chemotherapies[Title/Abstract]) OR Pharmacotherapy[Title/Abstract]) OR Pharmacotherapies[Title/Abstract]	306608
6	4 OR 5	1324840
7	"Neoplastic Cells, Circulating"[Mesh]	7617
8	(((((((((((((((((((((((((Circulating Cells, Neoplasm[Title/Abstract]) OR Neoplasm Circulating Cells[Title/Abstract]) OR Circulating Tumor Cells[Title/Abstract]) OR Cell, Circulating Tumor[Title/Abstract]) OR Cells, Circulating Tumor[Title/Abstract]) OR Circulating Tumor Cell[Title/Abstract]) OR Tumor Cell, Circulating[Title/Abstract]) OR Tumor Cells, Circulating[Title/Abstract]) OR Cells, Neoplasm Circulating[Title/Abstract]) OR Cell, Neoplasm Circulating[Title/Abstract]) OR Neoplasm Circulating Cell[Title/Abstract]) OR Circulating Neoplastic Cells[Title/Abstract]) OR Cell, Circulating Neoplastic[Title/Abstract]) OR Cells, Circulating Neoplastic[Title/Abstract]) OR Circulating Neoplastic Cell[Title/Abstract]) OR Neoplastic Cell, Circulating[Title/Abstract]) OR Tumor Cells, Embolic[Title/Abstract]) OR Cell, Embolic Tumor[Title/Abstract]) OR Cells, Embolic Tumor[Title/Abstract]) OR Embolic Tumor Cell[Title/Abstract]) OR Tumor Cell, Embolic[Title/Abstract]) OR Embolic Tumor Cells[Title/Abstract]) OR Embolism, Tumor[Title/Abstract]) OR Embolisms, Tumor[Title/Abstract]) OR Tumor Embolism[Title/Abstract]) OR Tumor Embolisms[Title/Abstract]	24301
9	7 OR 8	28397
10	response[Title/Abstract]	1626737
11	3 AND 6 AND 9 AND 10	187

Embase

Searched up to December, 2015

No.	Searches	Results
1	'lung cancer'/exp	253473
2	'pulmonary neoplasms':ab,ti	577

3	'lung neoplasm':ab,ti	329
4	'pulmonary neoplasm':ab,ti	335
5	'lung cancer':ab,ti	151630
6	'lung cancers':ab,ti	11788
7	'pulmonary cancer':ab,ti	1214
8	'pulmonary cancers':ab,ti	185
9	'cancer of the lung':ab,ti	2035
10	'cancer of lung':ab,ti	96
11	#1 OR #2 OR #3 OR #4 OR #5 OR #6 OR #7 OR #8 OR #9 OR #10	282050
12	'drug therapy'/exp	2078082
13	'drug therapies':ab,ti	4458
14	'chemotherapy':ab,ti	408742
15	'chemotherapies':ab,ti	6035
16	'pharmacotherapy':ab,ti	33422
17	'pharmacotherapies':ab,ti	3055
18	#12 OR #13 OR #14 OR #15 OR #16 OR #17	2231885
19	'circulating tumor cell'/exp	4929
20	'neoplasm circulating cells':ab,ti	1
21	'circulating tumor cells':ab,ti	4928
22	'circulating tumor cell':ab,ti	668
23	'neoplasm circulating cell':ab,ti	0
24	'circulating neoplastic cells':ab,ti	41
25	'circulating neoplastic cell':ab,ti	1
26	'embolic tumor cell':ab,ti	0
27	'embolic tumor cells':ab,ti	16
28	'tumor embolism':ab,ti	335
29	'tumor embolisms':ab,ti	11
30	#19 OR #20 OR #21 OR #22 OR #23 OR #24 OR #25 OR #26 OR #27 OR #28 OR #29	7013
31	'response':ab,ti	1997112
32	#11 AND #18 AND #30 AND #31	124

Cochrane Database

Searched up to December, 2015

No.	Searches	Results
1	MeSH descriptor: [Lung Neoplasms] explode all trees	5490
2	Pulmonary Neoplasms:ti,ab,kw (Word variations have been searched)	1091
3	Neoplasms, Lung:ti,ab,kw (Word variations have been searched)	6791
4	Lung Cancer:ti,ab,kw (Word variations have been searched)	10837
5	Pulmonary Cancer:ti,ab,kw (Word variations have been searched)	1212
6	Cancer of the Lung:ti,ab,kw (Word variations have been searched)	9051
7	Cancer of Lung:ti,ab,kw (Word variations have been searched)	10124
8	#1 or #2 or #3 or #4 or #5 or #6 or #7	12573
9	MeSH descriptor: [Drug Therapy] explode all trees	126693
10	Therapy, Drug:ti,ab,kw (Word variations have been searched)	144230

11	Chemotherapy:ti,ab,kw (Word variations have been searched)	38001
12	Pharmacotherapy:ti,ab,kw (Word variations have been searched)	5112
13	#9 or #10 or #11 or #12	240857
14	MeSH descriptor: [Neoplastic Cells, Circulating] explode all trees	102
15	Circulating Cells, Neoplasm:ti,ab,kw (Word variations have been searched)	364
16	Circulating Tumor Cells:ti,ab,kw (Word variations have been searched)	632
17	Circulating Neoplastic Cells:ti,ab,kw (Word variations have been searched)	125
18	Tumor Cells, Embolic:ti,ab,kw (Word variations have been searched)	7
19	Embolism, Tumor:ti,ab,kw (Word variations have been searched)	246
20	#14 or #15 or #16 or #17 or #18 or #19	993
21	response:ti,ab,kw (Word variations have been searched)	148967
22	#8 and #13 and #20 and #21	90

Web of science

Searched up to December, 2015

No.	Searches	Results
1	TS="Lung Neoplasms"	2184
2	TI=("Pulmonary Neoplasms" OR "Lung Neoplasm" OR "Pulmonary Neoplasm" OR "Lung Cancer" OR "Lung Cancers" OR "Pulmonary Cancer" OR "Pulmonary Cancers" OR "Cancer of the Lung" OR "Cancer of Lung")	81230
3	#2 OR #1	82445
4	TS="Drug Therapy"	29195
5	TI=("Drug Therapies" OR "Chemotherapy" OR "Chemotherapies" OR "Pharmacotherapy" OR "Pharmacotherapies")	105849
6	#5 OR #4	134228
7	TS="Circulating Tumor Cell"	597
8	TI=("Neoplasm Circulating Cells" OR "Circulating Tumor Cells" OR "Circulating Tumor Cell" OR "Neoplasm Circulating Cell" OR "Circulating Neoplastic Cells" OR "Circulating Neoplastic Cell" OR "Embolic Tumor Cell" OR "Embolic Tumor Cells" OR "Tumor Embolism" OR "Tumor Embolisms")	2744
9	#8 OR #7	2918
10	TS=response	2908137
11	#10 AND #9 AND #6 AND #3	5

Appendix 2. Assessment of publication bias

The results of publication bias

Comparison	P(Begg’s Test, continuity corrected)	P(Egger’s Test)
Baseline DCR	0.806	0.771
Baseline ORR	1	0.733
During chemotherapy DCR	0.734	0.781
During chemotherapy ORR	1	0.975

Group1 and 2 VS 3 and 4 progression rate	1	/
Group1 and 2 VS 3 progression rate	1	/
Group1 and 2 VS 3 nonresponse rate	1	0.914
OS baseline	0.308	0.121
OS duringtherapy	0.296	0.048
PFS baseline	0.296	0.351
PFS duringtherapy	1	0.111

NOTE. DCR: disease control rate; ORR: objective response rate; Group 1: patients who converted from CTC-negative to CTC-positive; Group 2: patients who were persistently CTC-positive; Group 3: patients who converted from CTC-positive to CTC-negative; Group 4: patients who were persistently CTC-negative; OS: overall survival; PFS: progression-free survival.
